# Isolation and characterization of halotolerant plant growth promoting rhizobacteria from mangrove region of Sundarbans, India for enhanced crop productivity

**DOI:** 10.3389/fpls.2023.1122347

**Published:** 2023-04-20

**Authors:** Pallavi, Rohit Kumar Mishra, Pramod Kumar Sahu, Vani Mishra, Hafiza Jamal, Ajit Varma, Swati Tripathi

**Affiliations:** ^1^ Amity Institute of Microbial Technology, Amity University, Noida, India; ^2^ Department of Microbiology, Indian Council of Agricultural Research – National Bureau of Agriculturally Important Microorganism, Kushmaur, Mau, Uttar Pradesh, India; ^3^ Centre of Science and Society, University of Allahabad, Prayagraj, Uttar Pradesh, India; ^4^ Nanotechnology Application Centre, University of Allahabad, Prayagraj, Uttar Pradesh, India

**Keywords:** halophilic bacteria, salt-affected soils, microbial diversity, mangrove, Sundarban

## Abstract

Halotolerant plant growth promoting rhizobacteria (PGPR) are beneficial microorganisms utilized to mitigate the biotic and abiotic stresses in plants. The areas of Sundarban mangroves of West Bengal, India have been reported to be rich in halotolerant microflora, yet major area remains unexplored. The present study, therefore, aims to map down the region-specific native microbial community potent of salt tolerance, plant growth promoting (PGP) activity and antagonistic activity against fungal pathogens. Bacterial samples were isolated from the saline soil of the Sundarban mangroves. A total of 156 bacterial samples were isolated and 20 were screened for their salt tolerance potential. These isolates were characterised using morphological, biochemical, and molecular approaches. Based on 16s rRNA sequencing, they were classified into 4 different genera, including *Arthrobacter* sp. (01 isolate), *Pseudomonas plecoglossicida* (01 isolate), *Kocuria rosea* (01 isolate), and *Bacillus* (17 isolates). The halotolerant isolates which possessed plant growth promoting traits including phosphate, and zinc solubilization, indole acetic acid production, siderophore, and ammonia generation were selected. Further, the effect of two halotolerant isolates GN-5 and JR-12 which showed most prominent PGP activities was evaluated in pea plant under high salinity conditions. The isolates improved survival by promoting germination (36 to 43%) and root-shoot growth and weight of pea plant in comparison to non-inoculated control plants. In a subsequent dual culture confrontation experiment, both these halo-tolerant isolates showed antagonistic activities against the aggressive root rot disease-causing *Macrophomina phaseolina* (Tassi) Goid NAIMCC-F-02902. The identified isolates could be used as potential bioagents for saline soils, with potential antagonistic effect on root rot disease. However, further studies at the physiological and molecular level would help to delineate a detail mechanistic understanding of broad-spectrum defence against salinity and potential biotic pathogen.

## Introduction

Major biotic and abiotic elements such as pathogen infections, salinity, drought, and extremely high temperatures can limit the growth and development of any crop and can significantly hamper its production on larger scale. Soil salinity is one of the major stress factors responsible for limiting the crop productivity across the globe. It impacts seed germination, plant growth and development, and reproductive success (Ke et al., 2020; Korenblum et al., 2020; Kumar and Sharma, 2020) eventually, leading to a decline in agricultural crop production across the globe. Increasing sea level due to global warming has been documented to increase soil salinity across the coastal areas thereby, casting deleterious effects on crops under the changing climate conditions (Tripathi et al., 2022). Growing crops in saline soil could be a viable option under current scenario with limiting land resources and increasing food demand, provided, crops can withstand mild to moderate salinity stress (Canfora et al., 2014; Xiaoqin et al., 2021). Considering reclamation of the saline soil is a complex process, use of beneficial microbes adapted to high salinity conditions provide viable option for improving crop production in salinity affected area.

Plant growth promoting rhizobacteria lessen the impact of biotic and abiotic stresses both directly and indirectly and can boost plant growth and crop output (Albdaiwi et al., 2019). Halotolerant PGPR reduce salinity damages in various crops wherein salt stress is significantly reduced which in turn improves agricultural output in terms of plant health and productivity even in saline alkaline soil (Etesami and Beattie, 2018; Numan et al., 2018; Kapadia et al., 2022; Khumairah et al., 2022; Reang et al., 2022). These halotolerant PGPR alleviate salinity stress by numerous mechanisms like enhanced water uptake capacity, accumulation of osmolytes (glutamate, glycine betaine, proline and soluble sugars), increased antioxidant level, synthesis of ACC deaminase, exoploysaccharides production and ionic homeostasis (Ha-Tran et al., 2021). By generating siderophore to sequester iron, solubilizing phosphorus, and by fixing nitrogen, they can directly enhance plant uptake of nutrients from its surrounding and enhance crop productivity (Etesami and Beattie, 2018) in soils with fertility and severe salinization problems (Khumairah et al., 2022). Additionally, phytohormones like indole acetic acid (IAA) produced by them influence the plant growth (Glick, 2014). Indirect plant growth promotion by these PGPR, on the other hand, happens when they reduce or stop plant damage brought on by pathogenic organisms including bacteria, fungus, and nematodes (Compant et al., 2005).

Indeed, over the years the coastal and saline regions including the Sundarban mangroves have turned out as a natural source of beneficial microbes adapted to high salinity levels. Sundarban mangroves are reported to have a unique, diverse, and rich indigenous microflora which are yet largely unexplored (Mitra, 2020). Given the distribution and diversity of these rhizobacteria that are unique to the mangrove region, justifies the need for research to learn about the region-specific native microbial community, characterise and identify it for effective crop development in stressful environments. Therefore, the major goal of this work was to explore rhizobacterial isolates from the Sundarban mangroves saline soil which can stand to be potential candidates for alleviating salt stress and augment plant productivity. In line with this objective, we isolated, screened, and characterized halotolerant PGPR from the Sundarban mangrove’s saline soil. The isolates were identified using 16 S rRNA sequencing and tested *in vitro* for plant growth promotion activities and salt stress tolerance at different levels of salinity. Twenty bacterial isolates could potentially tolerate salt stress and have significant plant growth promoting activity. Additionally, the isolates were examined for antagonistic activities against phytopathogen *Macrophomina phaseolina* (Tassi) Goid Strain NAIMCC-F-02902, which revealed 11 isolates exhibited antagonistic activity against it*. In-planta* studies of two most potential isolates GN-5 and JR-12 showed promising growth promoting activity against different salt concentrations in *Pisum sativum* L. which shows high sensitivity to water stress due to salinity.

## Materials and methods

### Sampling site

The soil samples were collected in triplicates from five sites in Sundarban mangrove locations in West Bengal, India (21°94’5.0”N and 88°89’58”E) during the summers (June–July 2015; Sites details: **Supplementary Table S1**). The plant root sections with soil, to a profundity of 15–30 cm from the rhizospheric region, immediately adjacent to the roots of the mangrove species *Heritiera fomes, Dipterocarpus retusus* Bl., *Exoecaria agallocho* L., *Acanthus ilicifolius* L. and *Bruguiera gymnorhiza* were collected and kept in a polythene bag at room temperature till further processing.

### Preliminary soil analysis

The chemical and physical characteristics of the collected soil samples were analysed. The standard methods were used to analyse the soil texture, and pH (Estefan et al., 2013). The electrical conductivity of the soil was measured as per the protocol described in Smith and Doran (1996) with slight modification. Briefly, soil sample (100 g) was taken in 200 mL distilled water and stirred well for a period of 30 sec. The solution was allowed to settle for 10 min and the EC was measured in this suspension at room temperature. Elemental analysis was performed by outsourcing at Directorate of Soybean Research Institute Indore, Madhya Pradesh, India (**Supplementary Table S1**).

### Isolation of bacteria

For bacterial isolation, 10g of each soil sample was dissolved in 90 ml of 0.85% saline. 100 µL samples from successive serial dilutions (10^−3^, 10^−4^ and 10^−5^) were spread on Nutrient agar and other selective media *viz.*, Yeast mannitol extract agar, King’s B, Soil extract agar, Trypticase soya agar plates and incubated at 37 ± 2°C till the appearance of bacterial growth.

### Halotolerance assay

Nutrient agar plates supplemented with various concentrations of NaCl at 0% (0 M), 5% (0.85 M), 10% (1.71 M), 15% (2.56 M) and 20% (3.42 M) were inoculated with uniform concentration of inoculum (OD_600_ = 0.5) from all the isolates and incubated at 37 ± 2°C for 7 days (Ramadoss et al., 2013) (**Supplementary Table S2**).

### Morphological characterization

For the morphological characterization, twenty screened halotolerant isolates were inoculated on nutrient agar plates and incubated at 37 ± 2°C for 24 h in incubator. The characteristics such as shape, size, margin, elevation, texture, and opacity were observed and recorded. The microscopic characterization by Gram’s staining and motility test were also performed (**Supplementary Table S3**).

### Biochemical characterization

The screened halotolerant isolates were tested for catalase activity by observing the bubble production (Reiner, 2010). For oxidase activity, p-amino dimethyl aniline oxalate, an oxidase reagent, was applied on the surface of the test bacteria (Shields and Cathcart, 2017). For Methyl Red test and VP test, nitrate broth and MR-VP broth were made, inoculated with 24 h old culture, and incubated at 37 ± 2°C for 24 h (McDevitt, 2009; Ferdous et al., 2013). For the TSI test, the triple sugar iron agar was inoculated with a 24 h old bacterial culture and incubated for 24 h at 37 ± 2°C (Cappuccino and Sherman, 2005). Simmon’s agar slants were prepared and inoculated with bacterial culture before being incubated at 37 ± 2°C for 24 h to test the citrate activity (McDevitt, 2009; Ferdous et al., 2013). Carbohydrate fermentation test to determine the ability to ferment a carbohydrate substrate with the production of gas and acid was conducted using trypticase 10 g L^−1^, sodium chloride 5 g L^−1^, phenol red 0.018 g L^−1^, and sugar (Mannose, Fructose, Sucrose, Xylose, Galactose) 5 g L^−1^ containing broth inoculated with 24 h old culture (Rahman et al., 2013) (**Supplementary Table S4**).

### Molecular characterization

#### Isolation of genomic DNA, 16S rRNA region sequencing, and phylogenetic analysis

The genomic DNA of screened halotolerant bacterial isolates were extracted using DNA extraction kit as per standard procedure (Mobio). PCR was used to amplify the 16S rRNA isolates with the universal primers 27 F (CATCTCAGTGCAACTAAA) and 1492 R (CAGGAAACAGCTATGAC). The PCR analysis employed 36 cycles of denaturation for 1 min at 94°C, annealing for 1 min at 54°C, and extension for 2 min at 72°C. The initial heating process lasted for two mins at 95°C. After that, the final conservatory phase was performed for 5 mins at 72°C. 15.3 mL of water, 3 mL of buffer (100 mM Tris-HCl, pH 8.3; 500 mM KCl), 1.8 mL of MgCl_2_, 0.6 mL of a combination of dGTP, dATP, dCTP and dTTP at concentration of 10 mM, and 3.0 mL of every primer (20.0 pmoles/mL) were included in the reaction mixture (Orhan and Gulluce, 2015). The PCR-amplified products were examined using gel electrophoresis on 1% agrose gel with 0.5 mg/ml ethidium bromide and a 1kb DNA molecular weight indicator, and their results were recorded using a gel certification system (Bio-Rad). Following the manufacturer’s recommendations, they were purified using the SV Gel and PCR Cleanup System (Bio-Rad). PCR products were sent to Macrogen (http://www.macrogen.com) for sequence analysis. The sequence data of all the isolates were analyzed and the results were submitted to the NCBI GenBank sequence database.

#### Evolutionary analysis by maximum likelihood method

The evolutionary history was calculated using the Maximum Likelihood approach and the Tamura-Nei model (Tamura and Nei, 2015). It displays the tree with the uppermost log probability (−7331.69). By first utilising the Neighbor-Join and BioNJ algorithms to a matrix of pair wise distances were estimated (Tamura-Nei model) and then by selecting the topology with the best log likelihood value, automatically generated starter trees for the heuristic search were produced. Branch lengths were stated in terms of the number of substitutions per site, and the tree was scaled. 30 nucleotide sequences were examined in this investigation. MEGA X [2] was used to carry out the evolutionary analysis.

### *In-vitro* assessment of direct PGP traits of bacterial isolates

Screened halotolerant bacterial isolates were studied for direct plant growth promoting traits.

#### Phosphate solubilization efficiency

Pikovskaya’s agar (PKA) medium was used to screen the bacteria, and tri-calcium phosphate (TCP) was utilised as a source of phosphate (Pikovskaya, 1948). Bacterial isolates were spotted on PKA plates after 24 h and incubated (37 ± 2°C) for one week. Bacterial colonies were surrounded by halo zones. The ability of the bacterial isolate to solubilise TCP on Pikovskaya’s (agar) medium was evaluated qualitatively. The colony and halo zone width were used to calculate the phosphate solubilization index.

The colony zone diameter was calculated by using the following formula of Edi et al. (1996).


, Phosphate Solubilizing Index (SI)=(CD+ZD)/CD

Where CD= Colony Diameter and ZD= Zone Diameter

To measure soluble phosphate *in vitro*, soluble phosphate availability in Pikovskaya’s broth supplemented with 0.5% TCP was utilised. The flasks in triplicates were incubated for 7 and 15 days (at 37 ± 2°C) on a rotatory shaker at 180 rpm before being centrifuged for 10 min at 10,000 rpm. The quantity of soluble phosphate that was available in the culture supernatant was measured using the Phospho-molybdate method (Watanabe and Olsen, 1965).

#### Production of indole acetic acid

Production of indole acetic acid was carried out using the Bricks technique (Brick and Bostock, 1991). In an incubator shaker, 10 ml of nutrient broth supplemented with 1% L-tryptophan was inoculated with freshly full-grown cultures and kept at 37 ± 2°C (48h) at 120rpm. At room conditions, cultures were centrifuged at 10,000 rpm (15 min). Salkowski reagent (2% 0.5 M FeCl_3_ in 35% perchloric acid) was added after 2 mL of supernatant was pipetted out. To develop the colour, two drops of orthophosphoric acid were also added, and the mixture was left in the dark. After two h, the visual density was recorded at 530nm. The standard plot of IAA was used to calculate the concentrations of IAA.

#### Production of siderophore

The Chrome Azurol Sulfonate (CAS) method was used to perform the siderophore production assay for qualitative analysis (Pérez-Miranda et al., 2007). The CAS medium was made according to Schwyn and Neilands (1987). Each bacterial culture was spot inoculated onto a CAS agar plate and incubated for 48 h (37 ± 2°C).

#### Zinc solubilization assay

The analysis of *in-vitro* zinc solubilization was carried out by employing plate assay. All bacterial isolates were tested for their capacity to solubilize insoluble zinc compounds, such as Zinc Oxide (ZnO). Single colonies that had grown for 24 h were spot inoculated on zinc medium plates amended by 1% ZnO (Sharma et al., 2012). For seven days, these plates were kept at 37 ± 2°C in the dark. Clear zones surrounded the colonies in zinc solubilizing isolates. These bacterial zonesdiameter was recorded.

#### Production of ammonia

The bacterial isolates were cultured in 10 ml of peptone broth and incubated at 37 ± 2°C (for 48h). Nessler’s reagent (0.5 mL) was added to the bacterial culture after incubation. Ammonia production was identified by the colour transition from brown to yellow (Cappuccino and Sherman, 2005).

### *In-vitro* assessment of indirect PGP traits by production of hydrolytic enzymes

#### Amylase activity

Bacterial cultures were screened for amylase enzyme production using a starch hydrolysis test (Shaw et al., 1995). Pure isolated colonies were streaked on starch agar plates and incubated at 37 ± 2°C for 48h. Plates were flooded with Gram’s iodine to produce a rich blue colour indicating complex of starch-iodine complex. Absence of blue colour in the zone of degradation is the basis of the detection of an amylase enzyme producing isolate.

#### Cellulase activity

Bacterial isolates were screened for cellulase enzyme production by CMC plate agar method. After incubation of 72 h at 37 ± 2°C, the plates were flooded with an aqueous solution of 0.1 percent Congo red for 15min with a subsequent wash with 1M NaCl to see the hydrolysed zone (Apun et al., 2000). The cellulase activity by bacteria was observed as the clear zone formed around the bacterial colonies on CMC agar plates.

#### Xylanase activity

The isolates were examined for the presence of the xylanase enzyme. Pure bacterial cultures were independently transferred onto the solution (yeast extract 0.5 g L^−1^, MgSO_4_ 0.5 g L^−1^, NaNO_3_ 1 g L^−1^, K_2_HPO_4_ 1 g L^−1^, KCl 1 g L^−1^, glucose 1 gL^−1^, agar 17 g L^−1^) containing 5 g l^−1^ of Xylan. After 48hrs of incubation, xylanolytic bacterial isolates were tested to see if they could break down xylan by flooding them with Congo red solution (0.1 percent) for 30 min, followed by washing them with 5mol l^−1^ NaCl (Amore et al., 2013). All isolates chosen as having xylanase activity were those that produced a distinct halo surrounding the colonies.

#### Pectinase activity

Pectinase-producing bacteria were screened in pectin-containing media [gL^−1^: yeast extract 1g, ammonium sulphate 2 g, Na_2_HPO_4_ 6 g, KH_2_PO_4_ 3 g, citric pectin 5 g, Agar-20g (pH 4.00)]. The colonies that displayed clear zones after being flooded with 1% cetyltrimethyl ammonium bromide (CTAB) after 24h of incubation were identified as pectinase producers (Altan, 2004).

### *In-vitro* assessment of antagonistic activity against *Macrophomina phaseolina* (Tassi) Goid

#### Dual culture plate assay

Selected halotolerant bacterial isolates were tested *in-vitro* to assess their ability to inhibit the pathogenic fungus *M. phaseolina* (Tassi) Goid Strain NAIMCC-F-02902 using dual culture method (Khamna et al., 2009). The fungus is soil borne and causes charcoal rot and dry root rot in many economically important crop plants (Wrather et al., 2010). The fungal pathogen *M. phaseolina* used in this study was procured from ICAR-NBAIM ‘NBAIMCC’ culture collection facility. Bacterial isolates were streaked at one side of modified PDA plates (90 mm diameter; 50% PDA + 50% NA), 48 h prior to pathogen inoculation. Five-day-old fungal mycelial discs were positioned at the other end of modified PDA plates (1cm from the border) against the bacterial streak and incubated at 37 ± 2°C. Control plates were those without the test isolate. For 5 days, all plates were incubated at 37 ± 2°C. The zone of inhibition (in mm) was measured, and colony growth inhibition percentage was calculated using the following equation after incubation:


PI=(C−T)*100/C

Where, PI is the percentage of inhibition; C is the colony growth of the pathogen in control, and T is the colony growth of the pathogen in the dual culture (Wonglom et al., 2019). All isolates were tested in triplicates.

### Pot experiment assay

The effect of screened salt tolerant PGPR inoculation on plant growth was investigated in pea plant (*Pisum sativum* L.) grown in pots. Seeds of pea were sterilized with 0.1% Carbendazim (Bavistin) solution for 5 min, thoroughly rinsed with double distilled water and dried on a Whatman paper before inoculation on the germination medium. Various NaCl concentrations [(50, 100, 150 and 200 mM) corresponding to (5, 9, 14.8, 18 dS/m)] were examined for the impact of salt stress on seed germination. The concentrations of the salts used in the experiments (50, 100, 150, and 200 mM) correspond to three classes of soil salinity: slightly saline (50 mM or 5 dS/m), moderately saline (100 mM or 9 dS/m) and strongly saline (200 mM or 18 dS/m). Similarly, sterilized seeds were sown in pots (5 cm diameter x 12 cm height) containing 50 gm garden soil supplemented with required amount of nitrogen, phosphorus, and potassium. Each pot was regularly maintained with optimum moisture. Seeds were immersed in 10mL of bacterial suspension (24 h culture in nutrient broth with concentration 1×10^9^ CFU mL^−1^) as per treatment for bacterization, and partially dried for 1h in uncovered petri dishes in a laminar flow hood. The control seeds were kept in sterile distilled water and dried at the same conditions. 5 seedlings per pot were grown under optimal conditions (29°C/26°C Day/night temperature) under controlled environmental chamber with 10,000 lx light at 10 h/14 h day/night cycle. Salinity stress treatment was given to the 14-d old pea seedlings to compare the changes in their root-shoot growth parameters. For salinity stress, a set of pots were enriched with 60 ml of 200 mM NaCl (HiMedia Maharashtra, India) [equivalent to 14 mg NaCl per gram of soil, thus, maintaining soil salinity close to 18 dS/m (Flower and Colmer, 2015; Rani and Sharma, 2015; Stoleru et al., 2019). The treatments were (i) Control: no salt (ii) Salinity treatment: salt @200 mM NaCl (iii) Test bacteria + Salinity@salt 200mM NaCl. Plant samples were harvested two-weeks after salinity treatment to study the root-shoot traits. Each treatment consisted of three replicates. On 15 days after germination, the growth parameters *viz.*, root length, shoot length, fresh weights and dry weights were recorded.

### Statistical analyses

All the experiments were conducted in triplicates and the results obtained were expressed in terms of mean of three biological replicates ± standard deviation. The means were compared with the least significant difference (LSD) and the levels of significance were represented with the *P*-value significance level. The data was analysed by GenStat release version 14 Ed. (Rothamsted Experimental Station, Harpenden, UK).

## Results

### Physico-chemical properties of collected soil sample

**Figure 1:**
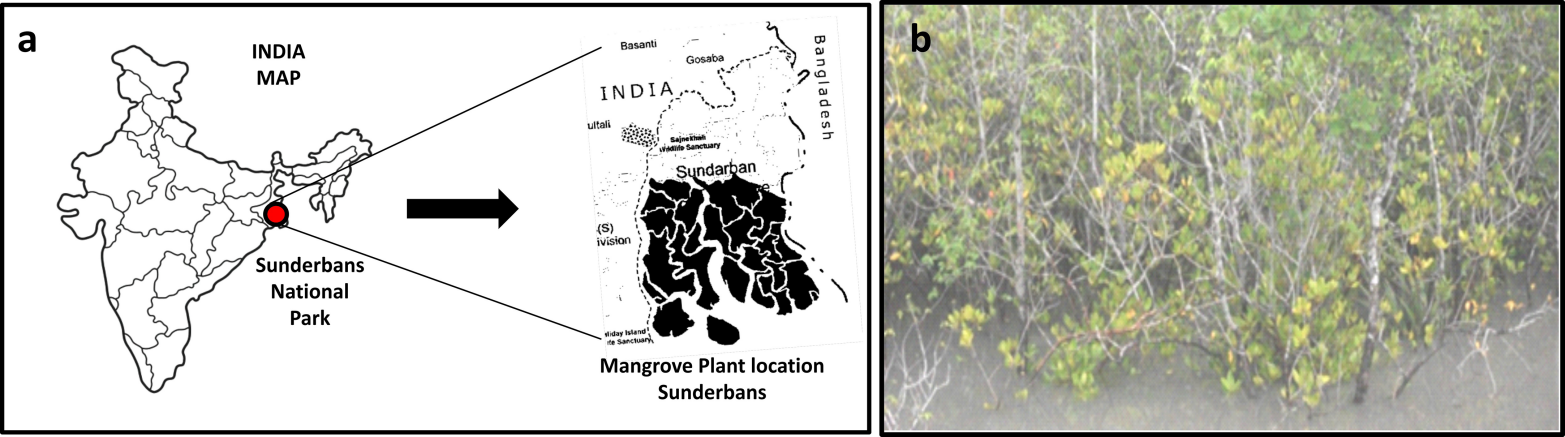
Study area for isolation of salt-tolerant bacterial isolates. **(A)** India map representing Sundarban area for the current study. **(B)** Mangrove plants at Sundarban, West Bengal, India.

In the present study, the soil samples were obtained from saline Sundarban mangrove regions in West Bengal, India (**Figure 1**; **Supplementary Table S1**). Rhizospheric bacteria from the rhizosphere of the mangrove plants, which varied in their growth and developmental stages, were isolated from soil samples. The soil had a sandy and loam texture. The highest soil organic matter level (0.45%) was found in Sundarban soil samples, with a pH range of 7.8 to 8.1. The EC values for soil salinity ranged up to 10.26 dS/m in the Kankara soil sample, which was regarded as extremely high and indicated a severe salty state that had an adverse impact on plant growth and development. **Supplementary Table S1** lists the physio-chemical characteristics of the soil samples.

### Isolation and halotolerance screening of bacterial isolates

156 bacteria were isolated and screened for the salt stress tolerance with NaCl concentrations of 5 (0.85 M), 10 (1.71 M), 15 (2.56 M) and 20% (3.42 M). 78 isolates could withstand up to 5%, 46 isolates could withstand more salt than 10%, 20 isolates could tolerate up to 15% NaCl. All these twenty isolates could grow at different pH ranging between 6 to 11 and were subjected to subsequent studies (**Supplementary Table S2**). Although the standard screening procedure involved substantially higher concentrations of salt stress (5–20%), the salinity level was kept up to 200 mM (18 dS/m) for plant growth assay with microbial association.

### Morphological characterization of screened bacterial isolates

Screened 20 halotolerant isolates were morphologically differentiated based on Gram’s staining, morphology, motility, colour, shape, margin, elevation, and endospore formation. Amongst all these 20 isolates, only GW-25 was a Gram −ve isolate, while the rest were Gram +ve and 3 isolates viz., KN-3, JT-1 and GW-25 were endospore negative. The only pink coloured isolate KN-3 was reported to be endospore negative, non-motile, and only cocci, while JT-1 remained the other non-motile isolate. The details of morphological characterization of all 20 isolates have been presented in (**Supplementary Table S3**).

### Biochemical characterization of screened bacterial isolates

The screened halotolerant isolates showed a diverse range of biochemical characteristics (**Supplementary Table S4**). Catalase activity was present in all the isolates except JT-1 and GW-25. All but three isolates namely GN-5, KN-3 and JR-12 had citrate activity. Of all the 20 isolates, 9 isolates (SD-2, KN-23, SV-4, SV-11, SV-15, SV-34, GW-13, GW-25, and HJ-7) were reported to be oxidase negative. The isolates tested negative for Methyl Red test except 2 isolates (JT-1, and HJ-7) while 9 isolates were found Voges-Proskauer positive (GN-14, GN-5, GN-25, SD-2, KN-3, SV-4, SV-11, SV-15 and Gw-13).

### Molecular identification of screened bacterial isolates

**Figure 2:**
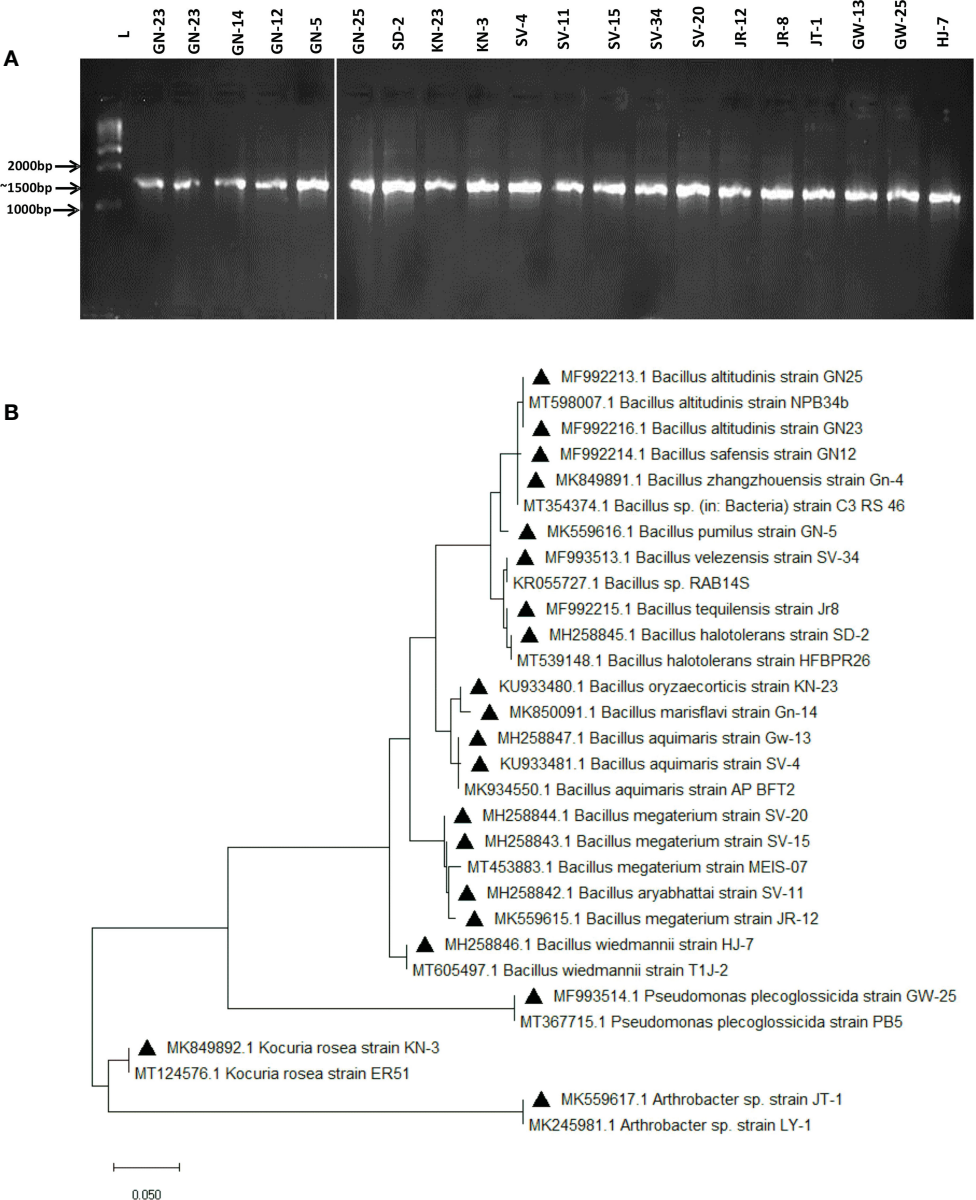
Molecular characterization of 20 halotolerant bacterial isolates. **(A)** PCR amplification of DNA fragments in rDNA in 20 selected salt tolerant isolates the expected DNA fragment of 16S is 1500 bp. (L) 100 bp DNA ladder (Promega). **(B)** Phylogenetic tree showing evolutionary relatedness among the isolates.

The screened 20 isolates were then identified using 16s rRNA sequencing. The bacterial isolates were successful in producing∼1500 bp amplification products (**Figure 2A**) and the BLASTN tool was used to analyse their DNA sequencing data. Most of the isolates were firmicutes, class Bacilli, and were identified as follows; *Bacillus altitudinis*(02), *B. marisfalvi, B. safensis, B. pumilus, B. halotolerant, B. oryzaecorticis, B. aquimaris* (03), *B. aryabhatti, B. megaterium* (03), *B. velezensis, B. tequilensis*, and *B. wiedmanni* were among the seventeen isolates. One was the actinobacteria phylum member *Kocuria rosea*, and the other was *Arthrobacter* sp. (01). *Pseudomonas plecoglossicida*, a proteobacteria species, was found as single isolates. **Supplementary Table S5** provides the accession numbers for all detected isolates and reference bacterium sequences.

### Evolutionary analysis by maximum likelihood method

The MEGA X 10.1.8 software application was used to create a phylogenetic tree and analyse phylogenetic relatedness using the greatest probability procedure (**Figure 2B**).

### *In-vitro* assessment of direct PGP traits of screened bacterial isolates

The screened 20 halotolerant isolates were assessed for the direct plant growth promoting traits viz., production of IAA, siderophore, ammonia and phosphate, and zinc solubilization potential.

#### Phosphate, zinc solubilization, ammonia and siderophore production

**Figure 3:**
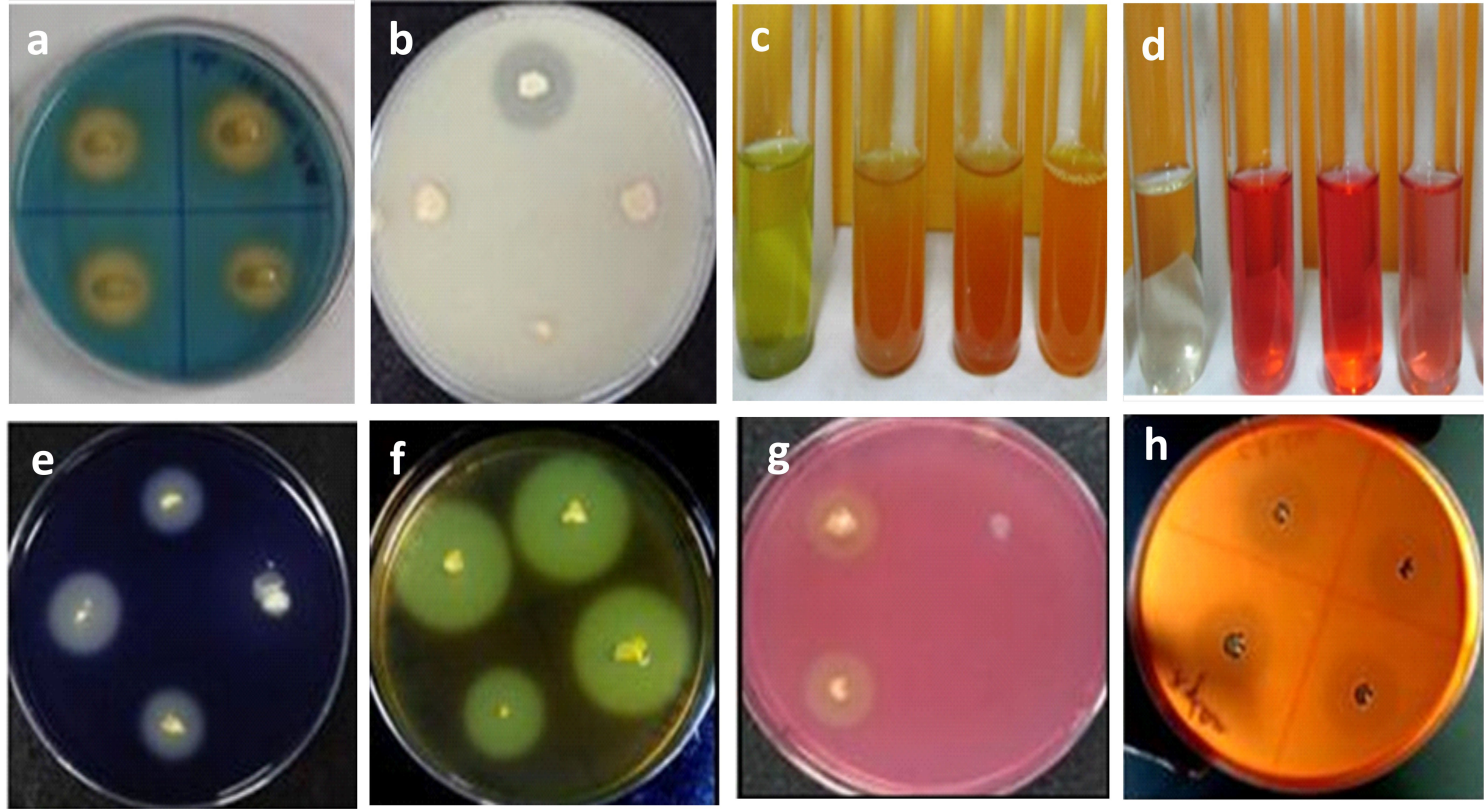
*In-vitro* assessment of direct and indirect plant growth promoting traits of halotolerant bacterial isolates: **(A)** Siderophore production; **(B)** Phosphate solubilization; **(C)** Ammonia production; **(D)** IAA production; **(E)** Amylase activity; **(F)** Pectinase activity; **(G)** Cellulose activity; **(H)** Xylanase activity.

On modified Pikovskaya agar plates, five of the 20 isolates were able to solubilize phosphate and produce clear zones (**Supplementary Table S6**). GN-5 was the most effective isolate at solubilizing phosphate (**Figure 3**). Six out of 20 isolates were able to create halo zones in the CAS-blue agar assay, indicating that they were able to produce siderophore, which was another method used to test for siderophore production (**Figure 3**, **Supplementary Table S6**). Bacilli strain SD-2 was regarded as a high-quality ammonia-producing bacterium. Five bacterial isolates were able to solubilize zinc (**Supplementary Table S6**).

#### Indole acetic acid production

Twelve bacterial isolates were able to produce high quantity of IAA out of the 20 isolates tested. 6 isolates out of 12 produced IAA levels more than 30µg/mL. The isolates that produced the most IAA were GN-5 and JR-12. It was determined that these two were closely related to *B. megaterium and B. pumilus* (**Figure 3**). The bacilli class member GN-25 was discovered to be a high-quality IAA generator (**Supplementary Table S6**).

### *In-vitro* assessment of indirect PGP traits by production of hydrolytic enzymes

Screened bacterial isolates were tested for indirect plant growth promoting traits by production of extracellular hydrolytic enzymes viz., pectinase, amylase, protease, and cellulose (**Supplementary Table S6**). 9 isolates (45%) produced cellulase (**Figure 3**), 03 (15%) produced xylanase (**Figure 3**), 03 (15%) produced pectinase (**Figure 3**) and 5 isolates (25%) produced amylase enzyme (**Figure 3**). 11 isolates showed no enzymatic activity, while 4 (20%) produced 3 extracellular enzymes, one (5%) produced 2 enzymes, and four isolates (20%) produced 1 enzyme (**Supplementary Table S6** and **Figure 3**).

### *In-vitro* assessment of antagonistic activity against *Macrophomina phaseolina* (Tassi) Goid

#### Dual culture plate assay

**Figure 4:**
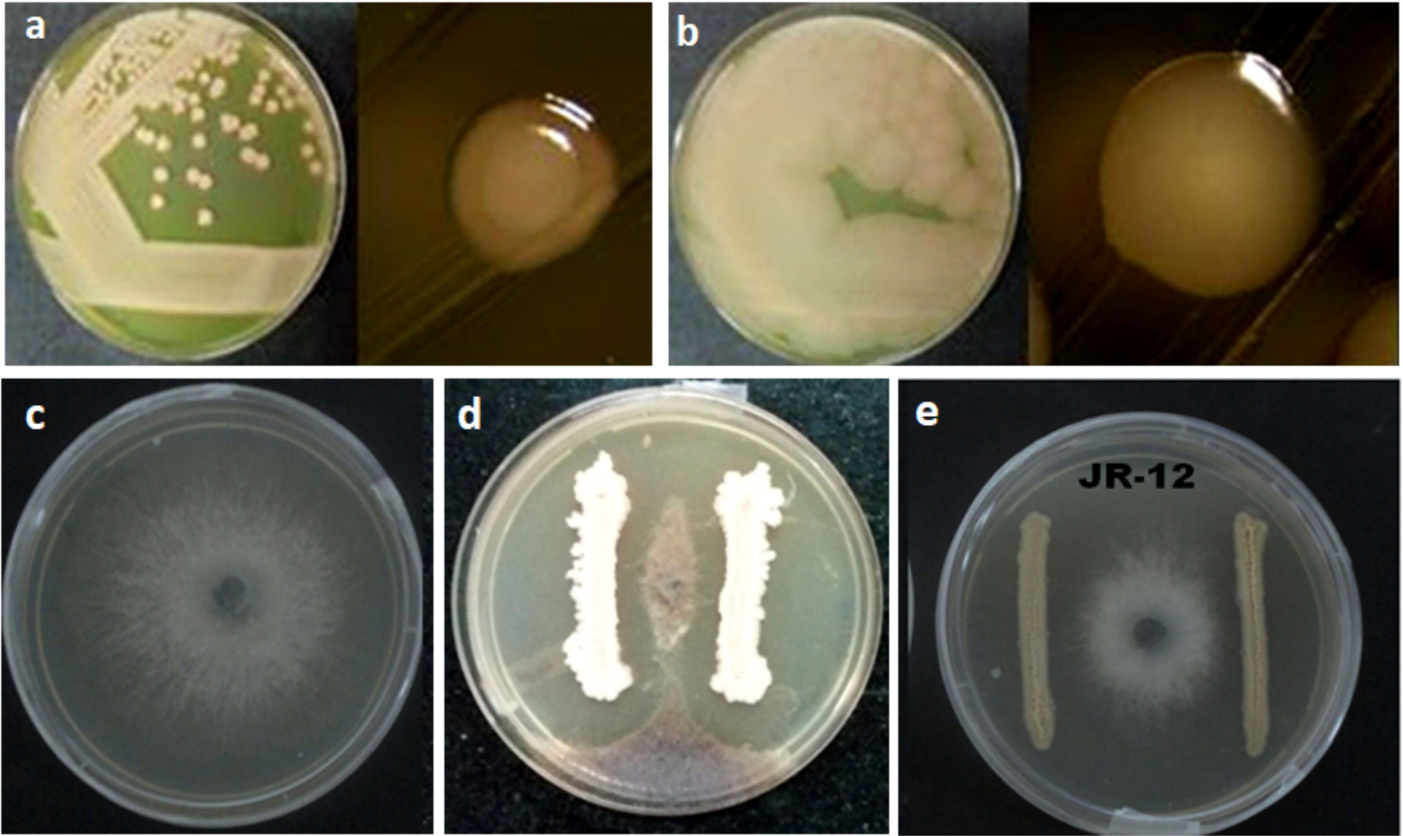
Antifungal activity of selected halotolerant plant growth promoting bacteria against *Macrophomina phaseolina* (Tassi) Goid. **(A)** GN-5 *Bacillus pumilus* MK559616. **(B)** JR-12 *Bacillus megaterium* MK559615. **(C)** Control *Macrophomina phaseolina* (Tassi) Goid. **(D)** Interaction of GN-5 *Bacillus pumilus* MK559616 and *M. phaseolina* (Tassi) Goid. **(E)** Interaction of JR-12 *Bacillus megaterium* MK559615 and *M. phaseolina* (Tassi) Goid.

In a dual culture plate assay, the halotolerant bacterial isolates; GN-4, 5, 12, 14, 25, SD-2, SV-15, 34, JR-8, 12 and GW-25 suppressed the growth of the mycelium against *Macrophomina phaseolina* (Tassi) Goid Strain NAIMCC-F-02902 (**Figure 4**). The highest level of antifungal activity which was ~36.66% was demonstrated by SD-2 among the other tested isolates. The other examined isolates showed no antifungal action against *M. phaseolina*.

#### Pot experiment assay for assessment of growth parameters

**Figure 5:**
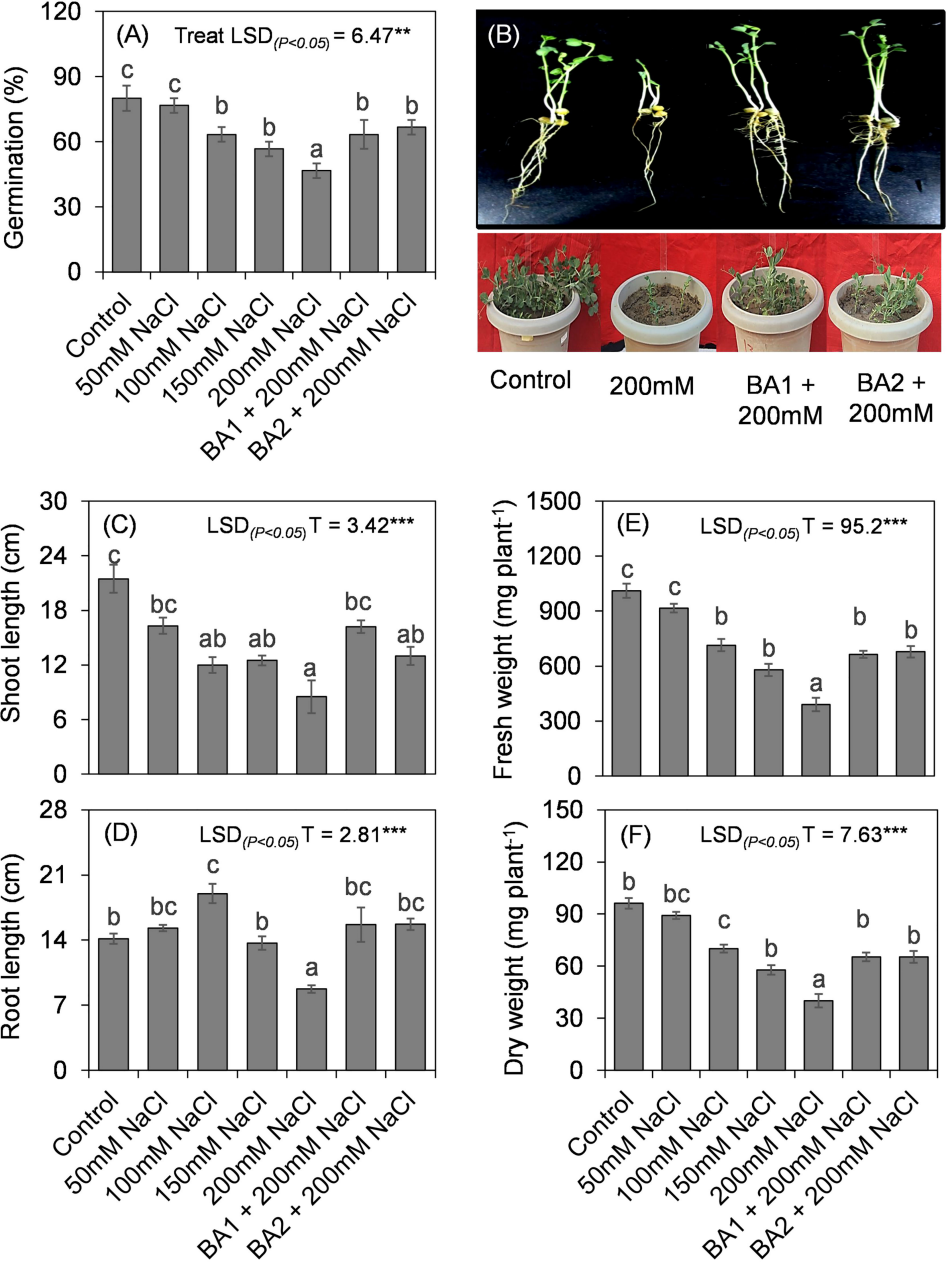
Effect of different concentrations of salt and selected halotolerant plant growth promoting bacterial isolates on *P. sativum* L. **(A)** Germination. **(B)** Pot experiment assay (2 weeks post treatment) [Treatments: C: Control; T1: 50mM; T2: 100mM; T3: 150mM; T4: 200mM; T5: JR 12 (*Bacillus megaterium* MK559615) + 200 mM; T6: GN 5 (*Bacillus pumilus* MK559616) + 200mM]. **(C)** Shoot length. **(D)** Root length. **(E)** Fresh weight. **(F)** Dry weight. Values are the means ± standard deviations of three experiments. Bars indicate ± SE (standard error of mean). Comparison of means was obtained from Tukey’s honest significance difference test. Means with the same letter are not significantly different at (*P*< 0.05). [Treatments: C: Control; T1: 50mM; T2: 100mM; T3: 150mM; T4: 200mM; T5: JR 12 (*Bacillus megaterium* MK559615) + 200 mM; T6: GN 5 (*Bacillus pumilus* MK559616) + 200mM].

Two isolates GN-5 and JR-12 of all the screened 20 halotolerant bacterial isolates showed majority of direct and indirect plant growth promoting traits (**Supplementary Table S6**) were selected for *in planta* assessment of growth parameters in *Pisum sativum* L. under saline conditions. The plants when inoculated with the two isolates could grow at all the salt concentrations [0 mM, 50 mM (5 dS/m), 100 mM (9 dS/m), 150 mM (14.8 dS/m) and 200 mM (18 dS/m)] however the PGPR treatment at 200 mM (18 dS/m) concentration outgrew the respective control treatment in growth parameters. JR-12 isolate increased germination by 35.7%, shoot length by 46%, root length by 79.3%, and fresh weight by 86.84%. When compared to the 200 mM control plants, isolate GN-5 increased germination percentage by 42.8%, shoot length by 52.9%, root length by 79.3% and fresh weight by 99.3% (**Figures 5A–F**).

## Discussion

In the present studies, 156 bacteria were isolated from soil samples collected from Sundarban mangrove, West Bengal, India. Saline soils have been shown to harbour native salt tolerant bacteria having potential role and efficiency in plant growth promoting activities (Yadav and Saxena, 2018), alleviating the effect of salt stress and helping in sustained crop production in salinity affected areas (Ramadoss et al., 2013; Shrivastava and Kumar, 2015; Sharma et al., 2021; Kumawat et al., 2022; Reang et al., 2022). Application of PGPR on different crops for increased crop productivity and salt stress alleviation have also been reported in previous studies (Mishra et al., 2010; Cherif-Silini et al., 2016; Albadaiwi et al., 2019; Tripathi et al., 2022). The soil property in the samples in our studies showed moderately alkaline nature with rich availability of potassium, nitrogen and iron, which might be due to the active participation of microbes in the bio-mineralization processes and biotransformation of minerals (Das et al., 2011). For the morphological and biochemical characteristics of these bacterial isolates, the colony characteristics were recorded in terms of shape, morphology, elevation, margin, motility, pH and enzyme production features. The isolates showed typical halophilic colony characteristics on media after 24 h. The similar observations were studied by Roohi et al. (2012). All the isolates were assessed for halotolerance by growing them in nutrient agar media amended with 0–20% (0–3.42 M) NaCl concentration at 37 ± 2°C. Twenty out of 156 isolates were screened to have high salt tolerance capability of up to 15% (2.56 M). Additionally, these salt-tolerant bacterial isolates could thrive at pH levels ranging from 6 to 11 (**Supplementary Table S2**), indicating their adaptation to the varying pH levels, besides salinity.

Phylogenetic analysis of these halotolerant isolates using 16S rRNA partial sequences revealed that the obtained isolates were highly diverse at the genus level. The majority of the isolates found were belonging to the class Bacilli *viz., B. megaterium, B. halotolerans, B. aquimaris, B. velezensis, B. altitudinis, B. tequilensis, B. oryzaecorticis, B. zhangzhouensis, B. marisflavi, B. safensis, B.aryabhattai, B.halotolerans, Kocuria rosea, B. wiedmannii, B. pumilus* and *Arthrobacter* most of which are well studied as beneficial PGPRs with growth promotion abilities under optimum and different abiotic and biotic stress conditions (Shukla et al., 2015; Vurukonda et al., 2016; Etesami and Beattie, 2018). Upadhyay et al. (2009) reported that the most halotolerant bacterial genera identified from rhizospheric soil under saline conditions belonged to the class Bacilli. Previous reports have shown presence of Bacillus-like halophilic bacteria from a variety of settings, including deep-sea hypersaline sediments and saline soils (Sass et al., 2008; Larose et al., 2013; Albdaiwi et al., 2019; Reang et al., 2022). *B. megaterium*, a salt-tolerant bacteria found in the Bhitarkanika mangrove in Odisha, has been able to grow well at >2M NaCl concentration (Mishra et al., 2011).

The direct and indirect plant growth promoting traits including inorganic phosphate and zinc solubilization, ammonia and IAA production, siderophore production, and antifungal effect against *M. phaseolina* were assessed in 20 selected isolates. The utilization of halotolerant PGPR with these PGP traits serves as a good strategy for the growth of salt sensitive plants (Etesami and Beattie, 2018; Gupta et al., 2021). P-solubilization assays of the screened 20 halotolerant bacterial isolates confirmed the phosphorus solubilization by the formation of clear zones, GN-5 isolate closely related to *Bacillus pumilus*, being the most effective. The measurement of zone of solubilization of isolates after 8 and 15 days were similar in range to that reported earlier (Zhu et al. (2011) and Reang et al. (2022). These results, however, have been reported to vary, based on nature, rate of release and the extent of spread of the metabolic substances responsible for solubilizing the phosphates and organophosphates in the vicinity of isolates (Timofeeva et al., 2022). PGPRs solubilize insoluble phosphates *via* numerous mechanisms like acidification by LMWOA, chelation and ion exchange (Etesami and Beattie, 2018), suppress the adverse effects of salt in salt-damaged soil and improve plant growth (Giri et al., 2004; Giri et al., 2007). P content and water use efficiency increased in tomato and wheat by inoculation of *Achromobacter piechaudii* and *B. aquimaris*, respectively (Mayak et al., 2004a; Rajput et al., 2013; Upadhyay and Singh, 2015) under saline soil conditions. Earlier, *Oceanobacillus picturae* isolated from the mangrove *A. marina* rhizosphere has been reported to mobilize 97% of this mineral (El-Tarabily and Youssef, 2011). Arthrobacter, Bacillus, Azospirillum, Phyllobacterium, and Vibrio solubilized P in various forms and increased it in both halophytes and glycophytes under salinity stress (Bashan et al., 2000; Yasmin and Bano, 2011; Nabti et al., 2010; Nabti et al., 2013). This increased P content in crops helps ameliorate the growth-restraining effects of salt induced stress. Subsequently, five isolates in the present study showed siderophore production indicating moderate sufficiency of iron in their habitat and environment (**Supplementary Table S6**, **Figure 3**). This also correlates with the highly halophilic nature of isolates which leads to very low to no siderophore production in such high salinity. Siderophores produced by these halotolerant bacteria in iron deficient environment help in the process of iron sequestration and solubilization (Sahu et al., 2021; Tripathi et al., 2022). Eleven of these 20 halotolerant isolates were positive for IAA production with JR-12 and GN-5 showing the highest production in the range of 35–45 μg mL^−1^. It was determined that these two isolates were closely related to *B. megaterium* and *B. pumilus.* This was observed to agree with the results of former studies reported by Bianco et al. (2006), Padder et al. (2017) and Nghia et al. (2017). However, these results were found to be very much lower in comparison to the range ~ 80 to 100 μg mL^−1^ (Nabti et al., 2017). IAA production being the most common trait of halotolerant PGPR (Dodd et al., 2010; Dodd and Pérez-Alfocea, 2012) has been reported to improve crop salt tolerance by various mechanisms like increasing the development of lateral roots and altering hormonal root–shoot signalling (Yang et al., 2009; Etesami and Beattie, 2018). IAA-producing salt-tolerant *Halomonas* sp. and *Sinorhizobium meliloti* have been reported to ameliorate the salinity stress impact in wheat and *Medicago truncatula* (Bianco and Defez, 2009; Tiwari et al., 2011), showing that modulation of IAA production by halotolerant PGPR plays crucial role in conferring salt tolerance. In the present study most of the isolates produced ammonia, with SD-2 and HJ-7 being highest ammonia producers. It has been suggested that the microbial cells fulfil the nitrogen demand by the host plants and promote growth parameters along with the biomass (Marques et al., 2010). Excessive production has also been reported to provide defence against phytopathogens through reduced pathogen colonization of the host plants and inhibition of further germination of fungal spores (Yadav et al., 2010). The salt tolerant isolates in the present study show significant antagonistic activity against *M. phaseolina* in the dual culture confrontation assay, as they show prominent extracellular hydrolytic enzyme (pectinase, amylase, cellulase and xylanase) production activities (**Supplementary Table S6**). These hydrolytic enzymes have prominent role in antagonistic activities due to their involvement in the degradation of cellulose, galactomannan or mannoprotein present in the fungal cell wall (Goswami and Deka, 2020). However, various biosynthetic pathways and involvement of several genes, the related regulatory sequences, and enzymes could be the determining factors for the variations in enzymatic production (Majeed et al., 2015). Shivanand and Jayaraman (2009), have earlier reported that *Bacillus aquimaris* strain isolated from the Kumta coast produce various extracellular enzymes. Many salt tolerant bacilli such as *B. subtilis, B. cereus, B. pumilus, Halomonas elongate, Halobacillus halophilus* have shown to antagonize numerous phytopathogenic fungi by the production of antibiotics, metabolites, extracellular enzymes, fungal cell wall degrading enzymes, HCN production, nutrient competition, and activating ISR (Sadfi-Zouaoui et al., 2008; Essghaier et al., 2009; Siddikee et al., 2010; Berrada et al., 2012; Ruppel et al., 2013; Goswami et al., 2014; Singh and Jha, 2016).

**Figure 6:**
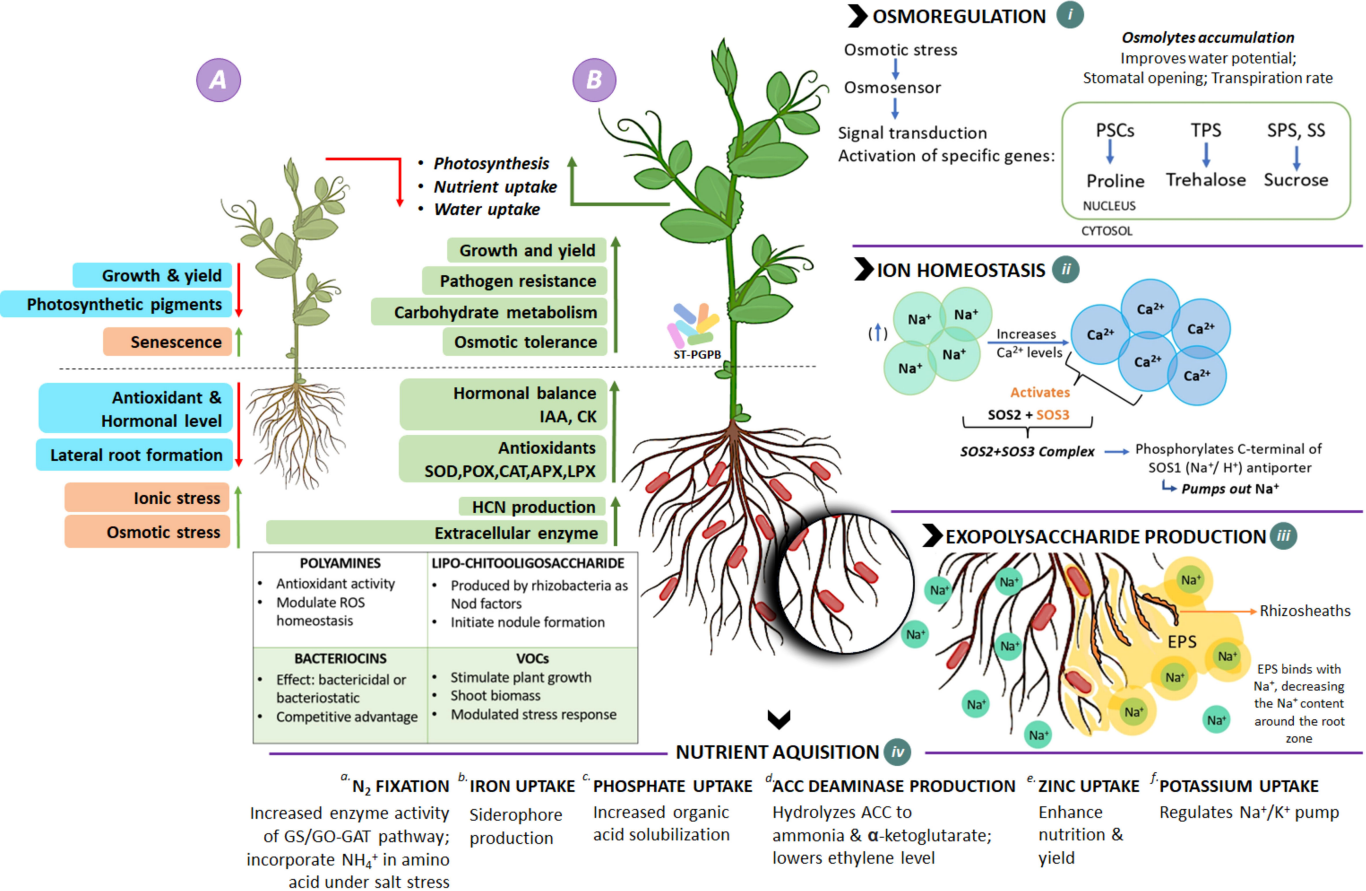
Schematic overview of role and mechanisms of salt-tolerant PGPR to alleviate salinity stress. **(A)** *Plant under salinity stress*: undergoes osmotic imbalance and ionic stress, which results in decreased lateral root formation inhibiting nutrient and water uptake, photosynthetic pigments, antioxidants, and growth and yield, ultimately leading to plant growth inhibition and premature senescence. **(B)** *Plant treated with PGPR*: Interaction with PGPR enhances overall plant health by mediating biochemical and physiological responses in plant cell during salt stress *via* various mechanisms. (i) Under such stress, PGPB promotes biosynthesis of osmolytes (proline, trehalose, surose etc.), balance osmosis; prevents cellular oxidative damage and improves membrane integrity (ii) *Ion homeostasis:* set of genes are upregulated, activating proteins SOS1, SOS2, SOS3 of SOS pathway, which pumps out excess Na+ through SOS1 antiporters. (iii) *Exopolysaccharide production:* EPS traps Na+, decreasing its amount in root zone, alters root structure by extensive rhizosheath formation. (iv)*Nutrient acquisition:* PGPB are known to escalate metabolic processes and provide nutrient availability by (a) N_2_ fixation: increased enzymatic activity of Glutamine synthetase/glutamine oxyglutarate aminotransferase (GS/GO-GAT) pathway which enables NH_4_^+^ incorporation in amino acids under saline stress condition; (b) Iron uptake: microbial induced metal chelation by siderophore production; (c) Phosphate uptake: increased organic acid solubilization; (d) ACC Deaminase: hydrolyses ACC to Ammonia & alpha-ketobutyrate, resulting in lowered level of ethylene in plants, inhibiting premature senescence; (e) Zinc uptake: increased nutrient uptake and yield; (f) Potassium: regulates Na+/K+ pump, balancing their concentration.

Two halotolerant isolates (GN-5 and JR-12) showed substantial plant growth promoting characteristics by increasing the germination percentage, shoot and root length, and fresh weight, dry weight of PGPR treated pea plants compared to the salt treatments. Majorly, the staple crops are glycophytes which get severely affected by soil salt concentration exceeding 200 mM (18 dS/m) and are unable to complete their life cycle (Munns and Tester, 2008; Flowers et al., 2015). In the current study we thus selected salinity level up to 200 mM (18 dS/m) in the pot experiments. Though, the increasing salinity stress led to decreased germination percentage in all the treatments, the maximum reduction was recorded in the plants with highest level, i.e., 200 mM (18 dS/m) salt concentration. Significant reduction was observed in all growth parameters by the increased salinity stress, but the PGPR treated plants showed maximum salt tolerance potential in terms of having the least reduction in all the parameters i.e., root and shoot length, and fresh and dry weights at 200mM (18 dS/m) salt concentration. The variations in germination percentage and subsequently in the growth parameters could be attributed to the deposition of Na^+^ and Cl^−^ ions in the tissues, eventually compromising the germination metabolism. Also, the salt stress decreases the water and osmotic potential, thereby, limiting the water uptake by the plant which leads to decline in germination and growth parameters under increased salinity conditions. This decrease in water potential also ends up in low cell turgor which has inhibitory effects on cell division and elongation and the growth of the plant gets delayed. The inhibitory effects of salinity stress on germination percentage and seedling growth have been well reported (Ahmad and Khan, 2010; Li et al., 2010; Shahid et al., 2012; **Figure 6A**). The relatively moderate reduction in PGPR treated plant growth parameters under salinity stress, could very well be related to the beneficial PGP functions of tested isolates. The positive impact of these selected isolates could also be related to multi-functional traits of the PGPR such as IAA production which leads to root initiation, cell division, increased root surface area, wherein root surface area and root architecture play the most crucial role (Dey et al., 2004; Gray and Smith, 2005; Alotaibi et al., 2022; **Figure 6B**) on plant growth under saline soil conditions. Certain osmolytes such as proline, trehalose, total soluble sugars are biosynthesised in response to osmotic stress which get accumulated in cytoplasm as compatible solutes. They get easily absorbed by plants; regulate water potential, stomatal opening and transpiration rate, balance osmosis, prevent cellular oxidative damage and improve membrane integrity (Sharma et al., 2019; Ghosh et al., 2021; **Figure 6B** i). These halotolerant PGPR also maintain ion homeostasis by activating Salt Overly sensitive (SOS) pathway, which triggers cascade of reactions (SOS1, SOS2, SOS3) mediating cellular signalling under saline conditions (Yang and Guo, 2018; Egamberdieva et al., 2019; Mishra et al., 2021; **Figure 6** ii). The activation of Na^+^/H^+^ antiporter pumps Na^+^ out of the cell. Homeostasis is also maintained in shoots by decreasing Na^+^/Cl^−^ accumulation in leaves, increased exclusion of Na^+^ from roots and enhanced activity of high affinity K^+^ transporters. These halotolerant PGPR have also been reported to modulate carbohydrate metabolism and transport directly implicating the source-sink relationship, photosynthesis, growth rate and biomass recollection (Ma et al., 2020; **Figure 6B**). Other mechanisms include production of exopolysaccharides, which trap Na^+^, decreasing its amount around root zone, alter root structure by extensive rhizosheath formation, stabilize soil structure and increase water and nutrient retention and further uptake (Fatima and Arora, 2021; **Figure 6B** iii). Apart from all these mechanisms, PGPR play their key role in facilitating nitrogen fixation by nodule formation; microbial induced metal chelation by siderophore production for iron uptake; phosphate uptake; ACC deaminase production, lowering ethylene levels that prevent premature senescence in plants; zinc and potassium uptake, balancing the Na^+^/K^+^ ratio (**Figure 6** iv). These properties exhibited by halotolerant PGPR significantly increase plant growth and yield in comparison to control under salinity stress which in turn upregulates hormonal levels, antioxidants, resistance to pathogens nutrient acquisition and plant-water relationship in plants (Kohler et al., 2009; Islam et al., 2016; Singh and Jha, 2016; El-Esawi et al., 2019; Fukami et al., 2018; Egamberdieva et al., 2019; López-Gómez et al., 2017; Sharma et al., 2019; Mishra et al., 2021; Kapadia et al., 2022; Tripathi et al., 2022; **Figure 6B**).

Therefore, the utilization of the plant growth-promoting rhizobacteria (PGPR) as an organic fertilizer additive can also help in reducing the use of chemical fertilizers in agriculture crop production (Kumawat et al., 2022). Individual plant responses, however, to different isolates with salt combination showed considerable variation and should be attributed to rhizospheric competencies. The significant increase in plant growth parameters upon application of these PGPR in comparison to control under salt stress individually and in combination clearly indicated the role of tested isolates in providing better nutrient flux and alleviation of salinity stress along with the biocontrol potential due to the additional presence of enzymatic activities. However, future studies using these isolates would be focussed to highlight the specific mechanisms allowing the host plant to thrive well under high salinity conditions.

## Conclusions

This extensive study has provided several bacterial salt tolerant isolates from saline environment of Sundarban mangrove. Characterization of these isolates gives insights in the plant growth promoting activity of specific isolates, which also impart salinity tolerance during pea seed germination and seedling growth. Phosphate solubilization, IAA production, production of siderophores and zinc solubilization are major important characteristics of the salt tolerant PGPR revealed in the present studies. Additionally, these isolates also showed biocontrol potential and resistance against the aggressive soil pathogenic fungi *M. phaseolina* causing root rot disease in legumes, due to the presence of enzymatic activities. This can be further used to exploit these isolates (GN-5 and JR-12) to make bio-formulations, targeting other crops facing salinity stress. Extensive field studies, however, should be required along with investigating the mechanisms of salinity tolerance induction at both, physiological as well as molecular levels.

## Data availability statement

The datasets presented in this study can be found in online repositories. The names of the repository/repositories and accession number(s) can be found in the article/**Supplementary Material**.

## Author contributions

P, RM and ST conceptualized and designed the experiments. P, PS and ST collected and analysed the data. P, PS, RM, VM, HJ and ST contributed to the manuscript. AV, RM and ST supervised the work. All authors contributed to the article and approved the submitted version.
